# The Clinical Suitability of an Artificial Intelligence–Enabled Pain Assessment Tool for Use in Infants: Feasibility and Usability Evaluation Study

**DOI:** 10.2196/41992

**Published:** 2023-02-13

**Authors:** Jeffery David Hughes, Paola Chivers, Kreshnik Hoti

**Affiliations:** 1 Curtin Medical School Curtin University Perth Australia; 2 Institute for Health Research The University of Notre Dame Australia Fremantle Australia; 3 School of Medical and Health Sciences Edith Cowan University Perth Australia; 4 Faculty of Medicine University of Prishtina Prishtina Kosovo

**Keywords:** pain assessment, clinical utility, sensitivity, specificity, immunization, accuracy, precision, PainChek Infant, infant, newborn, baby, babies, pain, facial, artificial intelligence, machine learning, model, detection, assessment

## Abstract

**Background:**

Infants are unable to self-report their pain, which, therefore, often goes underrecognized and undertreated. Adequate assessment of pain, including procedural pain, which has short- and long-term consequences, is critical for its management. The introduction of mobile health–based (mHealth) pain assessment tools could address current challenges and is an area requiring further research.

**Objective:**

The purpose of this study is to evaluate the accuracy and feasibility aspects of PainChek Infant and, therefore, assess its applicability in the intended setting.

**Methods:**

By observing infants just before, during, and after immunization, we evaluated the accuracy and precision at different cutoff scores of PainChek Infant, which is a point-of-care mHealth–based solution that uses artificial intelligence to detect pain and intensity based solely on facial expression. We used receiver operator characteristic analysis to assess interpretability and establish a cutoff score. Clinician comprehensibility was evaluated using a standardized questionnaire. Other feasibility aspects were evaluated based on comparison with currently available observational pain assessment tools for use in infants with procedural pain.

**Results:**

Both PainChek Infant Standard and Adaptive modes demonstrated high accuracy (area under the curve 0.964 and 0.966, respectively). At a cutoff score of ≥2, accuracy and precision were 0.908 and 0.912 for Standard and 0.912 and 0.897 for Adaptive modes, respectively. Currently available data allowed evaluation of 16 of the 17 feasibility aspects, with only the cost of the outcome measurement instrument unable to be evaluated since it is yet to be determined. PainChek Infant performed well across feasibility aspects, including interpretability (cutoff score defined), ease of administration, completion time (3 seconds), and clinician comprehensibility.

**Conclusions:**

This work provides information on the feasibility of using PainChek Infant in clinical practice for procedural pain assessment and monitoring, and demonstrates the accuracy and precision of the tool at the defined cutoff score.

## Introduction

Medical procedures, such as immunizations and other injections, heel pricks, venipunctures, and circumcision, are one of the commonest causes of pain in young children [1,2]. Pain associated with these procedures is referred to as procedural pain. Annually, 8 to 12 billion vaccinations are administered globally [1]. On admission to the hospital, children endure on average 4 procedures per day, but this number may be much higher [1,2]. Unfortunately, procedural pain often goes undertreated, and this is an issue because, even though the pain associated with needles may be considered short-lived, its consequences may be long-lasting. In the short term, inadequately managed pain related to needle procedures may lead to increased procedural time, use of restraint, increased pain and fear, dizziness and fainting, and the potential for injury [1]. Furthermore, depending on the procedure, the associated pain can also be an issue during the following days, as evidenced by Wood et al [3] who demonstrated the persistence of postvaccination pain at 4 days follow-up. Male circumcision, which is one of the most common surgical procedures, is also associated with subsequent persisting pain requiring treatment, as suggested by parents reporting on their child’s discomfort post procedure [4]. In the long term, poorly managed procedural pain may be associated with negative memories resulting in increased pain and fear of future procedures, and the need for additional analgesics to achieve the same effect, as well as delaying or avoiding procedures (eg, vaccine hesitancy) [1]. Additionally, evidence suggests there is a connection between the degree of acute pain exposure and ensuing cognitive, behavioral, and somatosensory outcomes in later life, including a stronger pain response when facing subsequent procedures [2,5].

Highlighting the need to make pain visible, Eccleston et al [2] emphasize the importance of assessing pain in children, including those who have not yet acquired the ability to self-report in whom behavioral scales should be used. In this regard, the evaluation of facial expressions in children is commonly used in various existing observational pain assessment tools and is a valid means of assessing pain [6]. However, evaluation of these facial expressions in clinical practice is done through direct observation, and this process is limited by the challenges of human decoding as well as inherent subjectivity issues [7,8]. For example, de Cassia et al [8] describe how facial expressions suggestive of pain are often not recognized by humans, and this depends on whether the assessor is a health professional or not, as well as their level of knowledge and education. These challenges can contribute to suboptimal identification and treatment of pain in infants. Unsurprisingly, exploring automated solutions that overcome limitations associated with the evaluation of infants’ facial expressions through direct observation has been an area of interest, as described by a number of studies focused on providing a solution to the problem via automatic recognition of facial expressions of pain [9]. However, there are currently no solutions used in clinical practice or at the point-of-care that have automated the process. In order to improve the current situation, the PainChek Infant app was developed, which is a point-of-care smart device technology-enabled application that uses automated facial analysis to identify 6 specific facial action units (AUs) to evaluate the presence and intensity of procedural pain in infants aged 1 month to 12 months [10]. This app was designed to improve the objectivity and accuracy of assessing procedural pain in infants and improve pain management for this vulnerable group. PainChek Infant App has been approved as a Class I medical device by the Therapeutic Goods Administration in Australia and received a CE mark in Europe “to assess and monitor pain procedural pain in infants aged 1 month to 12 months by health care professionals and laypersons” [11]. PainChek Infant demonstrated high levels of internal consistency and good to excellent interrater reliability when compared to both the neonatal facial coding system revised (NFCS-R) and the observer-administered visual analog scale (ObsVAS) [10].

This study was motivated by the fact that, while it is essential to establish the validity and reliability of new pain assessment tools, these features do not guarantee their adoption into clinical practice. Therefore, in this study, we assessed the accuracy and precision of the PainChek infant tool, together with the sensitivity and specificity for different cutoff scores using receiver operator characteristic (ROC) analysis. Clinician comprehensibility was evaluated using a standardized questionnaire. Additional feasibility aspects were evaluated based on comparison with currently available observational pain assessment tools for use in infants with procedural pain. We explore the feasibility and clinical utility aspects of PainChek Infant because they are also important criteria in evaluating new pain assessment tools given that they determine the usefulness and applicability of the tool in the clinical setting [12,13]. This is important, as the literature data indicates that these are often neglected during the evaluation of mobile health (mHealth) tools, including pain assessment tools [14,15].

## Methods

### Ethics Approval

This study was approved by the human research ethics committees of Curtin University (Approval Number: HRE2020-0315) and the Faculty of Medicine, University of Prishtina (Approval Number: No.3812/17).

### Study Design, Setting, and Inclusion Criteria

Complete details of the methodology used in the evaluation of the psychometric properties of PainChek Infant have been published previously [10]. In summary, the ability of PainChek Infant to accurately detect and quantify pain in infants undergoing routine vaccination was evaluated against the NFCS-R and the ObsVAS. Prerecorded videos of 40 infants were used from a purposely assembled digital library of 410 children (of which 329 infants) during April 2017-July 2018. The 40 infants were chosen from the pool of 329 using an electronic randomizer. From each randomly selected infant, four 10-second segments were extracted: Segment 0—Baseline (before any attempt to prepare the infant for the procedure was made [ie, while still in their parent’s arms]); Segment 1—Preparation (while the infant’s arm was prepared and swabbed); Segment 2—Immediately postvaccination (the painful part of the procedure [ie, within 10 s after needle insertion]); and Segment 3—Recovery (after the painful procedure [ie, between 10 s and 40 s after the needle insertion]) [10].

**Figure 1:**
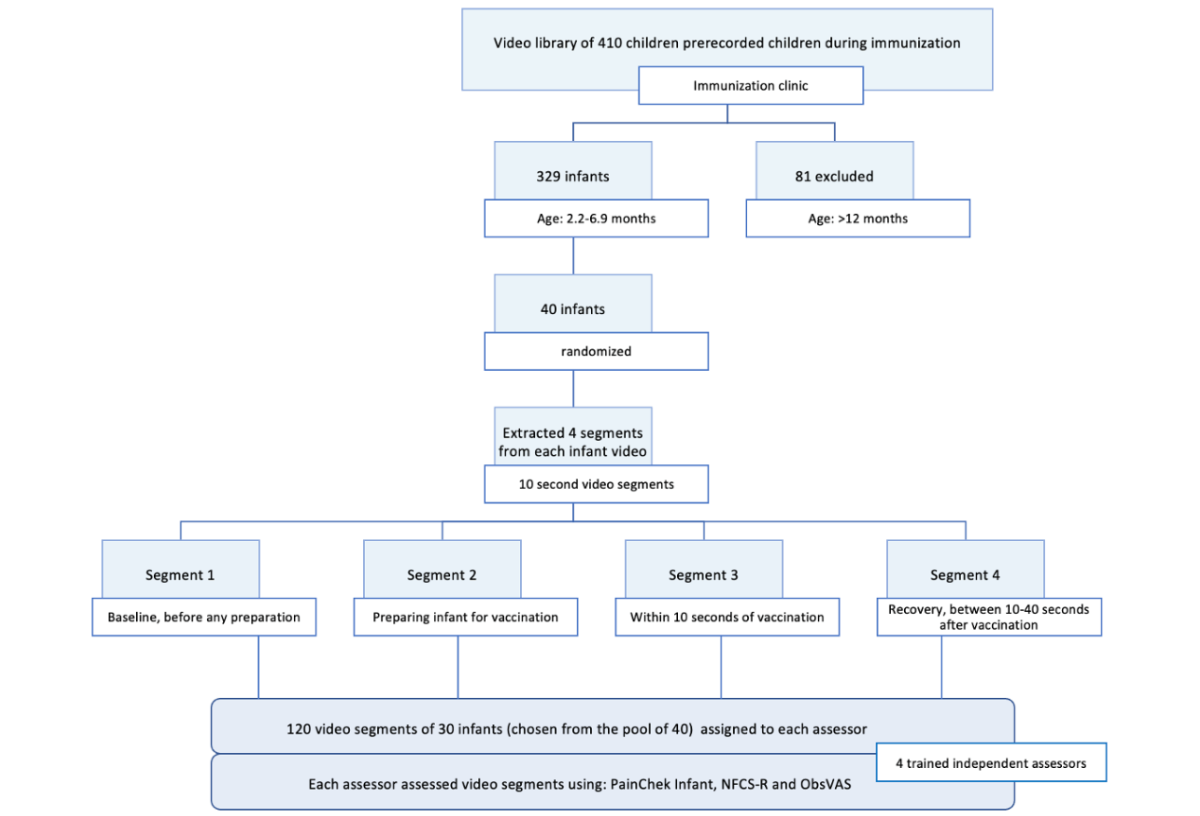
Flow diagram of the study design and setting. NFCS-R: neonatal facial coding system revised; ObsVAS: observer-administered visual analog scale.

Pain assessments were completed by 4 assessors independently, who were each assigned 120 video segments (ie, video segments of 30 infants) to assess from one of 2 testing session data sets. Each testing session included 1 clinically experienced (ie, pediatric nurse) and 1 clinically naïve assessor (ie, nursing student without pediatric clinical experience). Each assessor completed assessments using 3 pain assessment instruments (ie, PainChek Infant, NFCS-R, and ObsVAS). Details around the study design and setting have been illustrated in the flow diagram below (Figure 1).

We tested 2 modes of PainChek Infant analysis—Adaptive (predetermined minimum number of valid images, in this case, 1 image) and Standard (predetermined duration of video analysis, ie, 3 seconds). Assessors completed 2 rounds of assessments which were 4 weeks apart. They were blinded to the results of their fellow assessors. Pain assessments were completed using a single pain assessment tool at a time, to minimize recall bias. Furthermore, to avoid recall bias, the order of video segments was automatically and randomly assigned by the electronic data management system. Each testing session data set was assigned 2 assessors; 1 clinically experienced and 1 clinically naïve.

### Clinimetric Evaluation

An ROC analysis with sensitivity (true positive rate) and 100-specificity (false positive rate) for the PainChek Infant pain scores was conducted from the pain assessments completed as outlined above using NCSS Statistical Software (version 21; NCSS, LLC). Pain condition was determined based on pain score categories, that is, no pain (Segment 0: Baseline) and pain (Segment 2: Immediately postvaccination), with the prevaccination and recovery segments excluded from this analysis. The area under the curve (AUC) was determined with a null hypothesis using an upper one-sided AUC of 0.5 with empirical estimation. The test cutoff value was assigned as the point where sensitivity equals specificity [16]. For each cutoff score, sensitivity (true positive rate) with upper and lower 95% CI, specificity (true negative rate) with 95% CI, precision (positive predictive value), and accuracy were reported.

### Feasibility and Utility Questionnaire

A feasibility and clinical utility questionnaire was used to capture the assessors’ assessments of how easy each scale was to use and how well it performed (Table 1). The utility scale was developed by de Jong and colleagues [17], based on the criteria defined by Harris and Warren [18], and included 8 statements that were rated using a 5-point Likert Scale to assess the extent to which the assessor agrees with the statement. This evaluation was completed digitally after the assessors had completed the 2 rounds of pain assessments. The results of the survey are presented as mean scores and standard deviations.

**Table 1:** Feasibility and clinical utility questionnaire.

Statement	Level of agreement						
		1	2	3	4	5	
1. Provides information that is clinically useful	Clinically not very useful						Clinically very useful
2. Is it clear and easy to understand	Not clear and easy						Clear and easy
3. Is quick to apply	Very slow						Very quick
4. Is easy to apply	Very difficult						Very easy
5. Reflects the extent of procedural pain	Does not reflect at all						Reflects the extent well
6. Discriminates children with pain from children without pain	Does not discriminate at all						Discriminates well
7. Score is readily understood and supports decisions about pain management	Not readily understood and does not support decisions						Readily understood and supports decisions
8. Reflects procedural pain-specific features	Does not reflect procedural pain related features						Reflects procedural pain-related features

### Feasibility Aspects

In accordance with the “Guideline for selecting outcome measurement instruments for outcomes included in a Core Outcome Set,” PainChek Infant was assessed against the feasibility aspects set out in Textbox 1. This was done to answer the question, “Can the measure be applied easily in its intended setting, given constraints of time, money, and interpretability?” [16].

**Summary of all the feasibility aspects [16].**

**Feasibility aspects**

- Patient’s comprehensibility
- Ease of administration
- Interpretability
- Length of the outcome measurement instrument
- Completion time
- Patient’s mental ability level
- Ease of standardization
- Clinician’s comprehensibility
- Type of outcome measurement instrument
- Cost of an outcome measurement instrument
- Required equipment
- Type of administration
- Availability in different settings
- Copyright
- Patient’s physical ability level
- Regulatory agency’s requirement for approval
- Ease of score calculation

## Results

### Participants

A total of 40 White infants (24/40, 60% females) aged 2.2-6.9 (median 3.4, IQR 2.3-4.5) months undergoing routine immunizations were included in the study [10]. The 4 trained assessors comprised 2 experienced pediatric nurses (with 13 years and 16 years, respectively) and 2 final year Master of Nursing (Graduate-Entry) students, who were yet to complete any pediatric clinical placements. Three of the assessors were female (2 nurses and 1 student). The assessors were aged 33-37 (mean 35.5, SD 2.3) years.

### Clinimetric Evaluation

**Figure 2:**
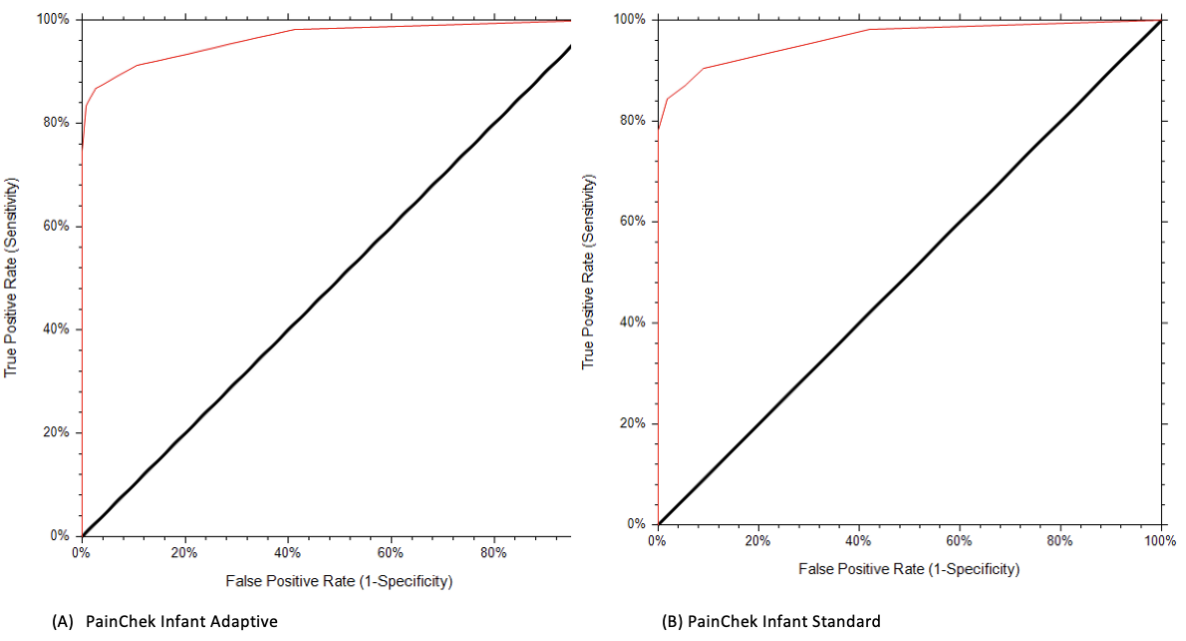
Receiver operator characteristic curves of video segments (0=baseline, 1=preparation, 2=vaccine, 3=recovery) for PainChek Adaptive (A) and PainChek Standard (B) modes.

To evaluate the diagnostic accuracy of tools 228 and 227 PainChek Adaptive and PainChek Standard assessments, respectively, were used to compare a baseline (no painful stimuli) and intervention (painful stimuli). A summary of AUC scores for each PainChek method (Adaptive and Standard) are presented in Table 2. Both PainChek Infant Standard and Adaptive modes demonstrate high accuracy with the AUC for both exceeding 0.9 (Figure 2).

Sensitivity (Se) and specificity (Sp) values for each cutoff score for each mode are summarized in Table 3. One of the frequently used criteria for the determination of the test cutoff value is the point where Se=Sp [19]. Therefore, based on those criteria a cutoff value of ≥2/6 was determined. At this cutoff, the sensitivity (Standard 0.904, Adaptive 0.912) and specificity (Standard 0.911; Adaptive 0.895) are equal for both PainChek Adaptive and Standard as shown in Table 3. Details of the accuracy and precision of PainChek Infant across a range of potential cutoff values are provided in Table 4. At a cutoff score of ≥2, accuracy and precision were 0.908 and 0.912 for Standard and 0.912 and 0.897 for Adaptive mode, respectively.

**Table 2:** The area under the receiver operator characteristic (ROC) curve.

Statistical item	PainChek Adaptive	PainChek Standard
Total N (Pain N)	228 (114)	227 (115)
Area under the ROC curve	0.966	0.964
SE	0.011	0.011
95% CI	0.936-0.982	0.934-0.980
*z* statistic	41.974	41.293
*P* value^a^	<.001	<.001

^a^Null hypothesis area under the curve 0.5 positive condition 2.

**Table 3:** Sensitivity and specificity calculated for different cutoff scores for PainChek Infant assessment methods.^a^

Cutoff score	PainChek Adaptive				PainChek Standard			
	Sensitivity	95% CI	Specificity	95% CI	Sensitivity	95% CI	Specificity	95% CI
≥0.0	1.000	0.968-1.000	0.000	0.000-0.032	1.000	0.968-1.000	0.000	0.000-0.032
≥1.0	0.983	0.938-0.998	0.588	0.492-0.679	0.983	0.939-0.998	0.580	0.483-0.673
≥2.0	0.912	0.845-0.957	0.895	0.823-0.944	0.904	0.835-0.951	0.911	0.842-0.956
≥3.0	0.868	0.792-0.924	0.974	0.925-0.995	0.870	0.794-0.925	0.946	0.887-0.980
≥4.0	0.833	0.752-0.897	0.991	0.952-1.000	0.844	0.764-0.905	0.982	0.937-0.998
≥5.0	0.746	0.656-0.823	1.000	0.968-1.000	0.783	0.696-0.854	1.000	0.968-1.000
≥6.0	0.658	0.563-0.744	1.000	0.968-1.000	0.704	0.612-0.786	1.000	0.968-1.000

^a^Pain was categorized as segment 0 (baseline) no pain, segment 3 (vaccination) pain.

**Table 4:** Precision and accuracy calculated for different cutoff scores for PainChek Infant assessment methods.^a^

Cutoff score			True positives		False positives		False negatives		True negatives		Sensitivity		Specificity		Precision		Accuracy		Sensitivity and specificity
**PainChek Adaptive**																			
	≥0.0	114		114		0		0		1.000		0.000		0.500		0.500		1.000	
	≥1.0	112		47		2		67		0.983		0.588		0.704		0.785		1.570	
	≥2.0	104		12		10		102		0.912		0.895		0.897		0.904		1.807	
	≥3.0	99		3		15		111		0.868		0.974		0.971		0.921		1.842	
	≥4.0	95		1		19		113		0.833		0.991		0.990		0.912		1.825	
	≥5.0	85		0		29		114		0.746		1.000		1.000		0.873		1.746	
	≥6.0	75		0		39		114		0.658		1.000		1.000		0.829		1.658	
**PainChek Standard**																			
	≥0.0	115		112		0		0		1.000		0.000		0.507		0.507		1.000	
	≥1.0	113		47		2		65		0.983		0.580		0.706		0.784		1.563	
	≥2.0	104		10		11		102		0.904		0.911		0.912		0.908		1.815	
	≥3.0	100		6		15		106		0.870		0.946		0.943		0.908		1.816	
	≥4.0	97		2		18		110		0.844		0.982		0.980		0.912		1.826	
	≥5.0	90		0		25		112		0.783		1.000		1.000		0.890		1.783	
	≥6.0	81		0		34		112		0.704		1.000		1.000		0.850		1.704	

^a^Sensitivity is the true positive rate; specificity is the true negative rate; precision is the positive predictive value; accuracy is the proportion correctly classified.

### Assessment of Feasibility Aspects

#### Patient Comprehensibility

This is not applicable in the case of PainChek Infant, as the tool is used by clinicians and care providers, including parents, and not by the patient.

#### Ease of Administration

**Figure 3:**
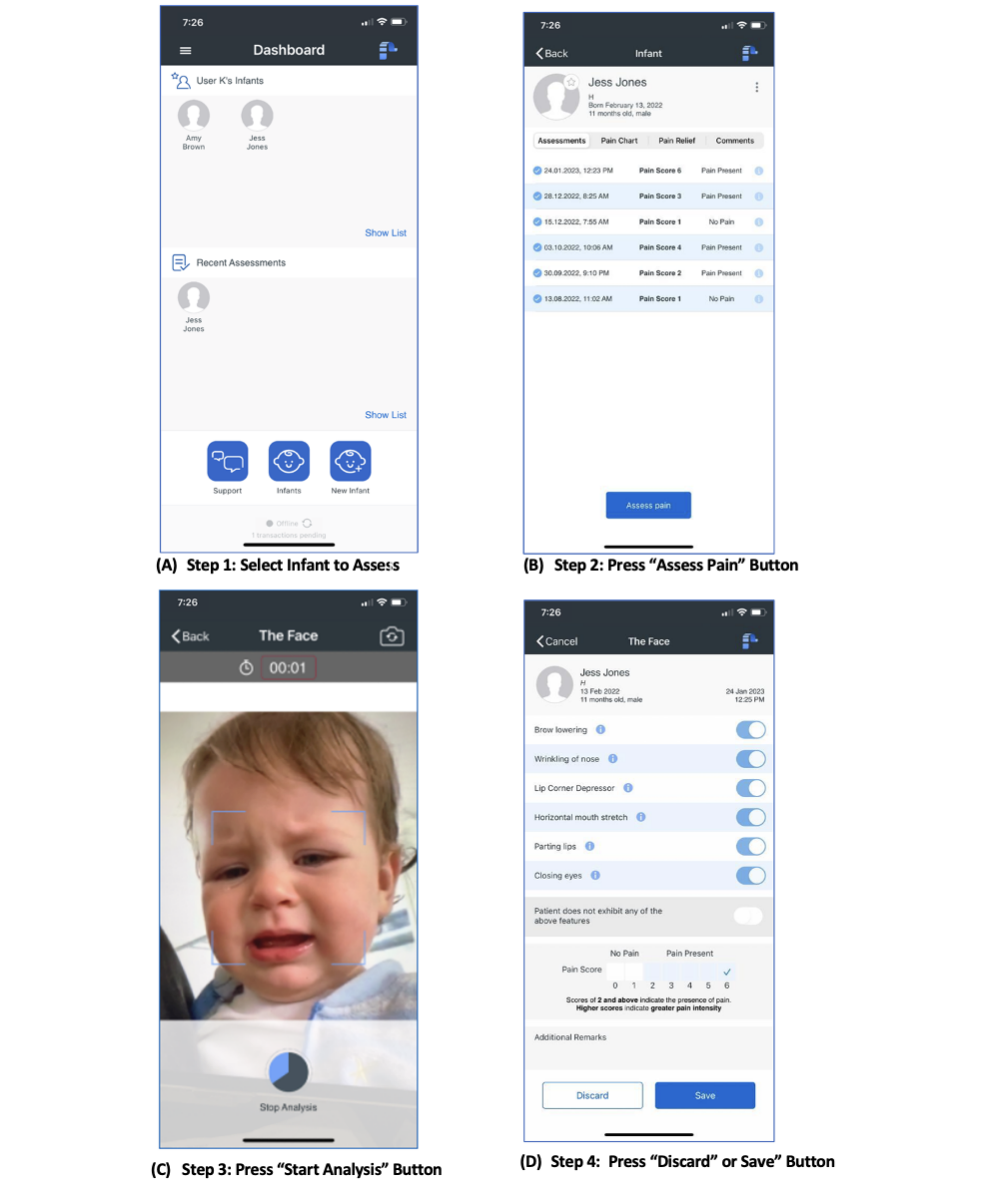
Key steps of the pain assessment process.

PainChek Infant requires the user to open the app after entering a 4-digit password-protected screen, select the infant to be assessed (Step 1), then press the “Assess Pain” button (Step 2) while pointing the device at the infant. The app automatically opens the device’s camera for the user. When the screen becomes active, the user then presses the “Start Analysis” button (Step 3). The app automatically completes an analysis of the infant’s face and records the presence or absence of the 6 pain-related AUs. After the analysis, the user is presented with a summary of the AUs present and a total pain score out of 6 for review. The user can then press the “Discard” or “Save” button (Step 4) to keep the assessment (Figure 3).

Hammal et al [20] stated, “a major challenge for manual FACS (facial action coding system) and BabyFACS is the extensive time involved in training expert coders and frame-by-frame annotation (or coding) from video.” FACS is labor-intensive. Training to criterion on the certification test for FACS can take months, and coding a single minute of video may require an hour or more [21]. Real-time coding for research or clinical use is not possible. Given these considerations, there has been great interest in developing approaches for the automatic recognition of FACS AUs [22]. With the view of addressing the abovementioned challenges, PainChek Infant was developed. Using artificial intelligence (AI), the smart device app takes 3 seconds to detect the presence or absence of 6 facial AUs indicative of the presence of pain in real time. The decision to use the software on a mobile device allows greater flexibility in what settings the tool can be used and for the user to position the device camera, avoiding possible obstruction of the face, and thus optimizing face detection and analysis. This overcomes a number of identified issues in using fixed camera systems. Further, software refinements have been incorporated to minimize analysis failure secondary to excessive head movement, and a “Retry” function is available if this does occur. Clinicians and caregivers are educated about infant pain and trained in the use of the device to ensure they use it under optimal conditions (eg, light, distance for infant, status of the infant), and they can interpret the results. Issues with lighting and head movement have been cited as limitations [9,20,22] to the use of automated facial analysis in clinical practice, hence why these measures have been put in place.

#### Interpretability

Interpretability was evaluated based on user feedback, clinimetric properties influencing interpretability, and the presentation of results for review by users, as detailed below.

##### User Feedback

**Figure 4:**
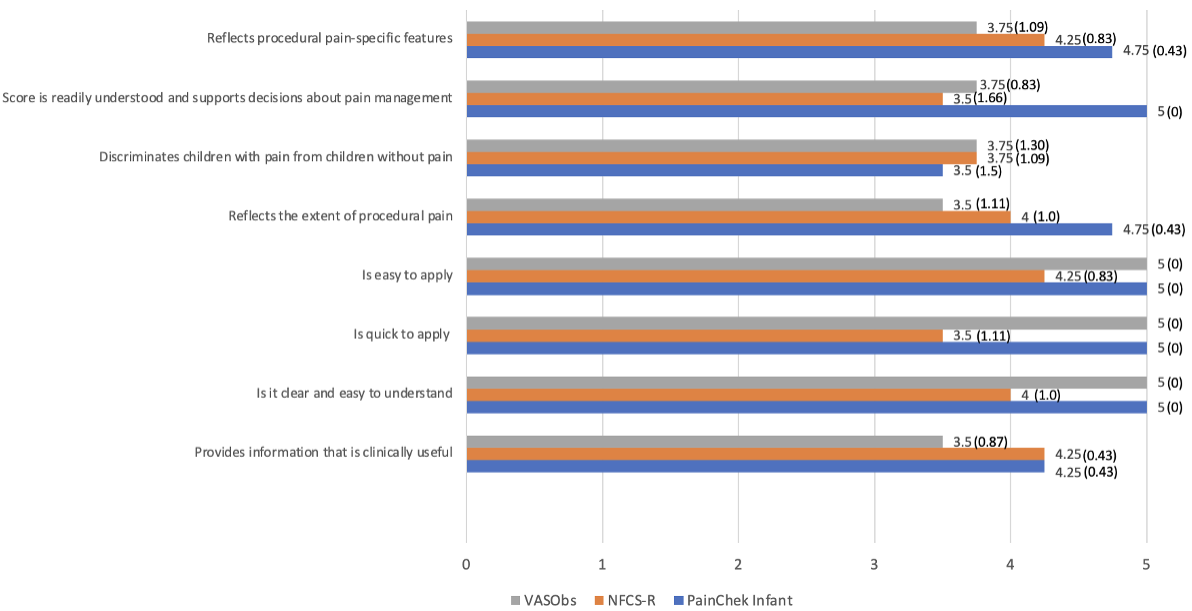
Assessor’s evaluations of the feasibility and clinical usability of PainChek Infant, the neonatal facial coding system revised (NFCS-R), and the observer-administered visual analog scale (ObsVAS). *Responses are rated on a 5-point Likert scale, where 0=strongly disagree and 5=strongly agree; results are presented as mean (SD).

The assessors involved in the study rated the feasibility and utility of PainChek Infant, NFCS-R, and ObsVAS. As can be seen from Figure 4 below, responses related to perceived interpretability were positive, with PainChek Infant generally outperforming the NFCS-R.

##### Clinimetric Properties

As outlined earlier, the ROC analysis demonstrated that the tool has high levels of accuracy and precision. Furthermore, PainChek Infant scores of 2 or above demonstrate the presence of pain with a high level of specificity and sensitivity.

##### Presentation of Pain Assessment Results

**Figure 5:**
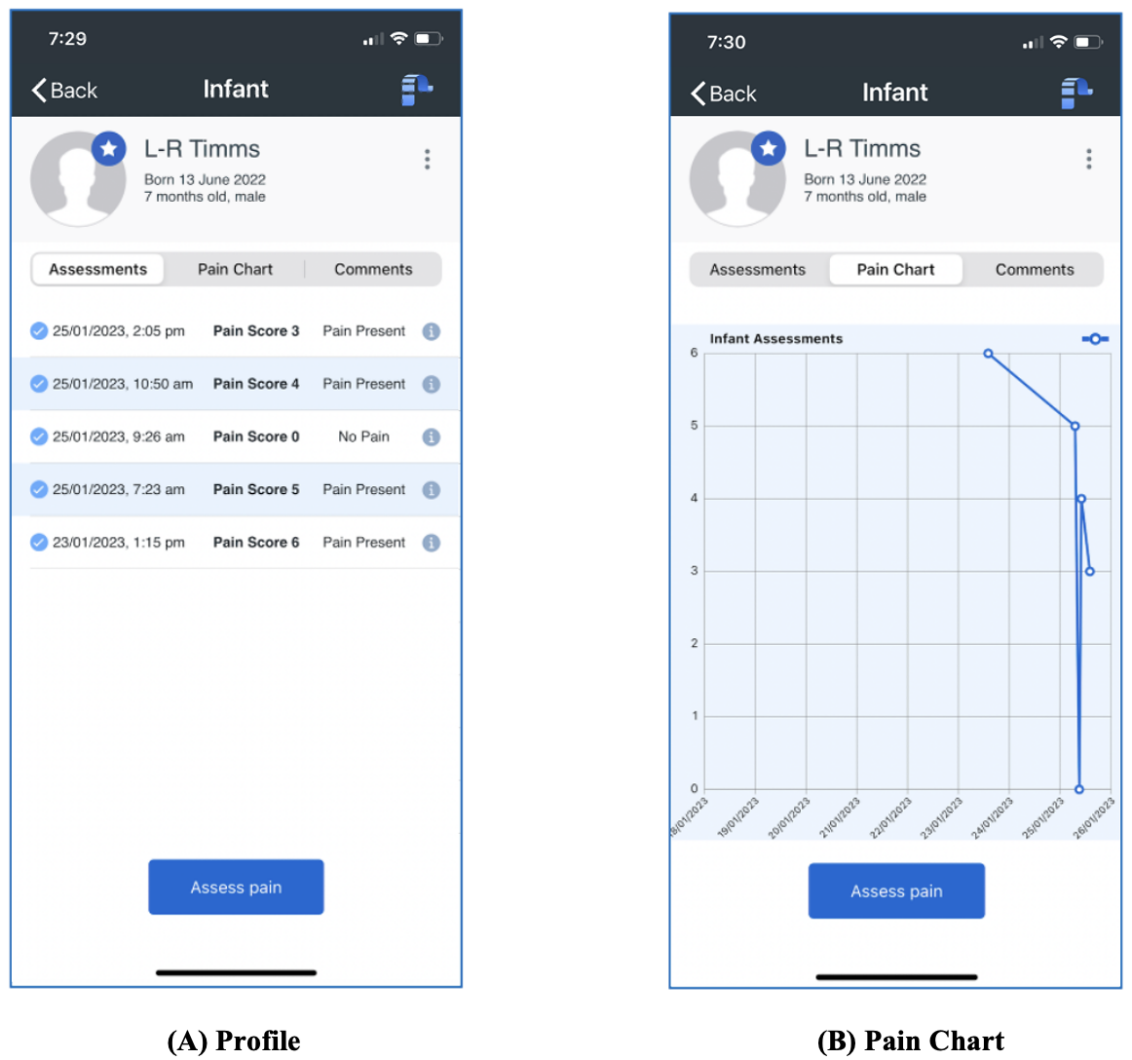
PainChek Infant pain monitoring. (A) Profile – recent pain assessments in reverse chronological order, (B) Pain Chart - graphical representation of pain scores versus time.

An interpretation of the score is provided to the user, that is, scores of 0 or 1 indicate no pain, and scores of 2 or above indicate pain. To further assist users in interpreting the results, pain assessments are presented in reverse chronological order in the infant’s profile (Figure 5A) and graphically over time (Figure 5B) for review.

#### Length of the Outcome Measurement Instrument (OMI)

The PainChek Infant pain assessment instrument contains 6 items in a single domain, the detection of which is fully automated. This is comparable to the NFCS-R, which contains 5 items (reduced from the 10 items that made up the original version of the tool) [23,24]. The Facial Action Summary Score (FASS), another unidimensional observational pain assessment tool, also includes 5 facial items [25]. The Faces, Legs, Arms, Cries, and Consolability (FLACC), multidimensional tool used for the assessment of procedural pain, has 5 domains; however, it uses an ordinal scoring system, such that the user is required to look for a variety of possible behaviors within each domain to determine absence or presence and intensity [26]. For example, for the face, a pain score of 0 indicates that there is no particular expression or smile; a score of 1 indicates occasional grimace or frown, being withdrawn or disinterested; and a score of 2 indicates that there is frequent to constant quivering chin and clenched jaw [26]. While the Modified Behavioral Pain Scale (MBPS), another multidimensional pain assessment tool used to assess procedural pain, only has 3 domains (facial expression, crying, and movements), the scale is comprised of 10 items, which are again scored ordinally (eg, Facial expression 0=definite positive expression [smiling], 1=neutral expression, 2=slightly negative expression [grimacing], and 3=definite negative expression [furrowed brow, eyes closed tightly]) [27].

#### Completion Time

The automated facial analysis takes 3 seconds to complete. Information on the time required to complete NFCS, including NFCS-R assessments at the bedside, is lacking. When video recordings are assessed, this is generally done on 10-second video segments, with coders scoring a varying number of epochs and then calculating a final score based on the number of assessments completed. These assessments can be completed at real speed, slow motion, or frame by frame depending on the coder’s choice, thus the time to complete could vary widely. In the study by Hoti et al [10], a pragmatic approach was adopted to the use of NFCS-R, where coders were asked to complete their assessment after a single viewing of each 10-second video segment, and after multiple viewings of the same segment, the results did not demonstrate any statistically significant differences. This approach also emulated real-world clinical practice settings in the context of the comparison with PainChek Infant. For the FLACC tool, it is recommended that patients who are awake be observed for 2-5 minutes and those who are asleep for at least 5 minutes or longer before scoring is completed [28].

#### Patient’s Mental Ability Level

PainChek Infant, like the NFCS-R, is administered by a clinician or care provider, and as such its use is not dependent on the mental ability of the patient for its administration. Among infants, it has been reported that pain-related facial expressions remain largely stable across the first year of life, although the intensity of pain-specific expressions may change [29]. Mercer and Glenn [30] assess facial expression responses in infants with developmental disabilities (DDs) (n=8) and typically developing (TD) infants (n=30) using the Maximally Discriminative Facial Movement Coding System during immunization. They reported that infants with DD expressed pain less clearly and for shorter periods of time compared to TD infants. No exclusion criteria for DD were applied in the recruitment of infants for the database used in the development of PainChek Infant; as such, it is probable that its algorithms were trained on images of both TD infants and those with DD. However, specific clinical studies to validate PainChek Infant's feasibility in assessing pain in infants with DD are required.

#### Ease of Standardization

The PainChek Infant assessment is standardized. The automated facial assessment takes 3 seconds to complete and is restricted to the detection of 6 pain-related facial AUs. The assessment is not dependent on the user’s interpretation of a child’s facial expressions, as is the case with the NFCS-R, therefore ensuring objectivity. Further, the facial expressions assessed represent defined muscle contractions or relaxations that are anatomically related, unlike facial items included in a number of multidimensional observational pain assessment tools whose descriptions are vague (eg, FLACC, MBPS) and may not reflect pain experience [31,32].

#### Clinicians’ Comprehensibility

As can be seen from Figure 4, responses related to comprehensibility were positive. The literature supports the findings around the NFCS and ObsVAS, and while the NFCS-R and PainChek Infant are similar in construct, additional data is required on their comprehensibility involving a larger cohort of clinicians.

#### Types of OMI

PainChek Infant is a unidimensional, observational pain assessment tool that uses automated facial recognition and analysis to identify the presence of 6 pain-related facial AUs. It has a similar construct to the NFCS-R and the FASS, both of which rely on an assessment of facial expressions by the user to evaluate the presence and intensity of pain [23-25].

#### Cost of an OMI

No information is currently available on the cost of PainChek Infant.

#### Required Equipment

PainChek Infant will operate on any iOS smart device (phone or tablet) capable of running iOS Version 14.0 (Apple Inc) [33]. To download the software, access to the internet is required; however, the device may be used at the point-of-care without internet connectivity.

#### Type of Administration

PainChek Infant is a software application that is administered at the point-of-care using a smart device that is equipped with a camera and processor.

#### Availability in Different Settings

PainChek Infant, as it is administered using a smart device, has the potential to be used across a variety of settings where procedural pain may occur, ranging from hospitals or day surgery centers to ambulatory care clinics, general practices, dental clinics, and the home care environment [34].

#### Patient’s Physical Ability

As PainChek Infant evaluates an infant’s face for the presence of facial expressions indicative of pain, PainChek Infant is advised to be used with caution on infants born with craniofacial birth defects and neuromuscular disorders, including but not limited to the following, considering that each will have varying degrees of clinical presentation: cleft lip and cleft palate, facial palsy, vascular birthmarks and hemangiomas, and hairy nevus [33].

#### Copyright

The copyright for the PainChek Infant tool is held by PainChek Ltd.

#### Regulatory Agency’s Requirement for Approval

PainChek Infant has regulatory clearance, including the Therapeutic Goods Administration, CE Mark, and UK MHRA, which allows its clinical use in Australia, Europe, the United Kingdom, Canada, Singapore, and New Zealand [4]. In the United States, based on the “Clinical Decision Support Software, Draft Guidance for Industry and Food and Drug Administration Staff,” issued September 27, 2019, (“CDS Draft Guidance”), PainChek Infant is not a medical device when used by health care professionals and will be marketed as a clinical decision support device [35].

#### Ease of Score Calculation

After completing the automated facial analysis, PainChek Infant provides a summary of the facial AUs detected and automatically calculates the pain score (Figure 3, Step 4).

## Discussion

### Principal Findings

In this study, we describe the clinimetric evaluation of PainChek Infant and determine its accuracy, precision, specificity, and sensitivity. We further expand to evaluate the feasibility aspects of its use. In doing so, we sought to address the following question: “Can the measure be applied easily in its intended setting, given constraints of time, money, and interpretability?” [16]. A positive answer to this question was important before attempting widespread implementation of the tool in clinical practice, something that the literature suggests has often been neglected in the development of other tools [14,15]. Here we provide further evidence related to the above, offering health care professionals and laypersons the necessary evidence to evaluate the implementation of the tool in clinical practice.

Evaluation of the usability of PainChek Infant was done in accordance with ISO 9241-11 of The International Organization for Standardization. According to these standards, usability is defined as the “extent to which a system, product, or service can be used by specific users to achieve specific goals with effectiveness, efficiency, and satisfaction in a specific context of use” [36], where effectiveness refers to the accuracy and completeness with which users achieve specified goals, in this case, the assessment of procedural pain.

While an increase in facial expression is used as a marker of intensity across both unidimensional and multidimensional observational pain assessment tools, there is an overlap between pain-related facial action and other emotions. Defining the cutoff score, therefore, provides the user with increased certainty that the facial expression observed is pain-related, thereby guiding toward pain relief. This is further supported by the high levels of accuracy and precision. It is worth mentioning that the cutoff threshold identified in this study is similar to that reported for the FASS using the FLACC as a comparator in postoperative pain [25]. The FASS and the NFCS-R are unidimensional tools, made up of 5 facial items. Based on achieving a combination of the highest specificity and sensitivity, Bringuier et al [25] reported a threshold of ≥2/5. In the Médecins Sans Frontières clinical guidelines, it is indicated that a score of ≥2 in the NFCS suggests serious pain [37].

PainChek Infant, which is a regulatory-cleared medical device, performed well across all evaluable feasibility aspects, including interpretability (with a cutoff score defined), ease of administration (based on assessor feedback), length of OMI (6 items), completion time (3 seconds), ease of standardization (ensured through AI-enabled facial analysis), clinician’s comprehensibility (clear and easily understood, cutoff score availability), type of OMI (point-of-care, digital assessment tool for procedural pain), required equipment (smart device), type of administration (point-of-care, automated), availability in different settings (used anywhere mobile devices can be used), and ease of score calculation (automated). The tool is intended to be used by health care professionals and caregivers; therefore, the feasibility aspects related to the “patient’s mental ability level” and the “patient’s physical ability” were evaluated with respect to the potential to influence facial analysis and considered limited, while its usefulness was not reliant on the “patient’s comprehensibility.”

The time needed to complete the pain assessment may be used as a measure of efficiency [38], which in the case of PainChek Infant is 3 seconds. In comparison, other tools commonly reported to be used for the assessment of procedural pain in infants take a longer time (eg, FLACC: 2 to 5 minutes; COMFORT: 2 minutes after establishing baseline heart rate and mean arterial pressure; MBPS: 5 seconds preprocedure and 15 seconds postprocedure) [27,39]. Further, it is not reliant on user observations, therefore eliminating the issue of potential user-related bias.

Satisfaction, which is the extent to which the user’s physical, cognitive, and emotional responses that result from the use of a tool meet the user’s needs and expectations, can be assessed in a number of ways, including interviews, focus groups, scales, and questionnaires [17]. In this case, we used the feasibility and clinical utility questionnaire, which, while acknowledging the limitation of the small sample size, demonstrated positive results.

It is important to acknowledge the limitations of the small cohort of assessors involved in the original study, and therefore, the need to repeat the feasibility and clinical utility questionnaires in a larger group of users. Another limitation is the fact that the evaluation of PainChek Infant was undertaken using video recordings of infants undergoing standard immunization rather than in a real-world setting. However, this method of validating pediatric pain assessment tools is common, allowing a larger number of assessors to be engaged in the validation process, as well as allowing multiple viewings, which is also considered a strength [14,27,40]. Furthermore, it should be emphasized that there may be an overlap between pain and non-pain–related distress, which can be caused by various situations, including hunger and restraint. This has also been acknowledged by other studies reporting on the validation of pain assessment tools in this population group [10,26,27]. As previously reported, the PainChek Infant tool, similar to compared tools used in this study, exhibited a clear change in facial expressions across all stages of the vaccination process, from baseline to recovery [10]. Furthermore, it is important to note that Kohut et al [41] have previously been successful in distinguishing facial expressions from non–pain-related distress for up to a minute following needle insertion. Nonetheless, to mitigate this issue, one should acknowledge that both PainChek Infant and NFCS-R have clinical utility when the source of pain is suspected or known.

In summary, mHealth solutions provide many benefits for health care professionals, including access to point-of-care, which has been shown to support better clinical decision-making and improve patient outcomes [42]. Yet, there is still reluctance among health care professionals to use them in clinical practice. To address this, there is a need for better standards and validation practices regarding mobile medical apps to ensure their proper use and integration into medical practice [42]. Therefore, this study offers further evidence to support the use of the world’s first point-of-care mHealth solution that assesses procedural pain in infants via AI-enabled analysis of facial expressions. At the same time, recognizing the need for further research related to its implementation into clinical practice.

### Conclusions

Careful evaluation of digital health solutions is a critical step prior to their widespread clinical implementation. This study provides further evidence in support of PainChek Infant’s accuracy, precision, feasibility, and usability as a regulatory-cleared tool for assessing procedural pain in infants, therefore offering solid grounds for its clinical practice implementation.

## Abbreviations

AI: artificial intelligence
AU: action unit
AUC: area under the curve
DD: developmental disability
FACS: Facial Action Coding System
FASS: facial action summary score
FLACC: Faces, Legs, Arms, Cries, and Consolability
mHealth: mobile health
MBPS: Modified Behavioral Pain Scale
NFCSR: neonatal facial coding system revised
ObsVAS: observer-administered visual analog scale
OMI: outcome measurement instrument
ROC: receiver operator characteristic
TD: typically developing
